# Benefit of Action Naming Over Object Naming for Visualization of Subcortical Language Pathways in Navigated Transcranial Magnetic Stimulation-Based Diffusion Tensor Imaging-Fiber Tracking

**DOI:** 10.3389/fnhum.2021.748274

**Published:** 2021-11-05

**Authors:** Ann-Katrin Ohlerth, Roelien Bastiaanse, Chiara Negwer, Nico Sollmann, Severin Schramm, Axel Schröder, Sandro M. Krieg

**Affiliations:** ^1^Center for Language and Cognition Groningen, University of Groningen, Groningen, Netherlands; ^2^International Doctorate for Experimental Approaches to Language and Brain (IDEALAB), University of Groningen, Groningen, Netherlands; ^3^Center for Language and Brain, National Research University Higher School of Economics, Moscow, Russia; ^4^Department of Neurosurgery, School of Medicine, Klinikum Rechts der Isar, Technical University of Munich, Munich, Germany; ^5^Department of Diagnostic and Interventional Neuroradiology, School of Medicine, Klinikum Rechts der Isar, Technical University of Munich, Munich, Germany; ^6^TUM-Neuroimaging Center, Klinikum Rechts der Isar, Technical University of Munich, Munich, Germany; ^7^Department of Diagnostic and Interventional Radiology, University Hospital Ulm, Ulm, Germany

**Keywords:** navigated transcranial magnetic stimulation (nTMS), diffusion tensor imaging fiber tracking, Action Naming, Object Naming, language mapping

## Abstract

Visualization of functionally significant subcortical white matter fibers is needed in neurosurgical procedures in order to avoid damage to the language network during resection. In an effort to achieve this, positive cortical points revealed during preoperative language mapping with navigated transcranial magnetic stimulation (nTMS) can be employed as regions of interest (ROIs) for diffusion tensor imaging (DTI) fiber tracking. However, the effect that the use of different language tasks has on nTMS mapping and subsequent DTI-fiber tracking remains unexplored. The visualization of ventral stream tracts with an assumed lexico-semantic role may especially benefit from ROIs delivered by the lexico-semantically demanding verb task, Action Naming. In a first step, bihemispheric nTMS language mapping was administered in 18 healthy participants using the standard task Object Naming and the novel task Action Naming to trigger verbs in a small sentence context. Cortical areas in which nTMS induced language errors were identified as language-positive cortical sites. In a second step, nTMS-based DTI-fiber tracking was conducted using solely these language-positive points as ROIs. The ability of the two tasks’ ROIs to visualize the dorsal tracts *Arcuate Fascicle* and *Superior Longitudinal Fascicle*, the ventral tracts *Inferior Longitudinal Fascicle, Uncinate Fascicle*, and *Inferior Fronto-Occipital Fascicle*, the speech-articulatory *Cortico-Nuclear Tract*, and interhemispheric commissural fibers was compared in both hemispheres. In the left hemisphere, ROIs of Action Naming led to a significantly higher fraction of overall visualized tracts, specifically in the ventral stream’s *Inferior Fronto-Occipital* and *Inferior Longitudinal Fascicle*. No difference was found between tracking with Action Naming vs. Object Naming seeds for dorsal stream tracts, neither for the speech-articulatory tract nor the inter-hemispheric connections. While the two tasks appeared equally demanding for phonological-articulatory processes, ROI seeding through the task Action Naming seemed to better visualize lexico-semantic tracts in the ventral stream. This distinction was not evident in the right hemisphere. However, the distribution of tracts exposed was, overall, mirrored relative to those in the left hemisphere network. In presurgical practice, mapping and tracking of language pathways may profit from these findings and should consider inclusion of the Action Naming task, particularly for lesions in ventral subcortical regions.

## Introduction

In the past decades, there has been a shift in allocation of language representation in the brain, from a focus on mostly cortical structures to an emphasis on the importance of the subcortical networks (Duffau, 2008; Dick et al., 2014), labeled as a “hodotopical approach” (Duffau et al., 2014). This change in emphasis is supported by the observation that damage to subcortical tracts may lead to even more severe, irreversible language loss than damage to cortical areas (Trinh et al., 2013; Duffau, 2014). As a consequence, in neurosurgical practice, increasing attention has been devoted to the preservation of subcortical language tracts. Intraoperatively, monitored and neuro-navigated resection of white matter in close proximity to these tracts is used to minimize post-operative impairments (Duffau et al., 2002, 2003; Duffau, 2005; Bello et al., 2007; Sanai and Berger, 2010).

Consequently, efforts have been made to better visualize the white matter tracts preoperatively in order to enhance surgical planning (Abhinav et al., 2014; Wende et al., 2020). Diffusion tensor imaging (DTI) is used as a means of structural depiction of subcortical white matter, where, in combination with fiber tracking, single tracts can be identified that are relevant for language functions. For this tracking, information in the form of regions of interest (ROIs) is needed for seeding, that is, start and/or end points at the cortical level that are connected by the subcortical tracts to be visualized. These seeds can be based on anatomical landmarks, but due to individual variation and anatomical shifts through tumor growth, this approach is not favored (Southwell et al., 2016). Functional seeding, using cortical areas that have been proven to be functionally involved during the execution of a language task, is considered superior to using anatomical seeds (Schonberg et al., 2006; Kleiser et al., 2010; Sollmann et al., 2016; Negwer et al., 2017b; Sanvito et al., 2020). These functional seeds can be acquired through functional magnetic resonance imaging (fMRI), but, more recently, have also been obtained using navigated transcranial magnetic stimulation (nTMS) mapping. Cortical areas in which errors are elicited during picture naming under nTMS disturbance, are considered positive language areas, and defined as ROIs to visualize tracts during subsequent tractography (Sollmann et al., 2015a, 2016, 2018a,b; Raffa et al., 2016; Negwer et al., 2017a, 2018; Ille et al., 2018).

This method of nTMS-based DTI fiber tracking has been successfully refined over the past few years. Optimal tracking parameters concerning Fractional Anisotropy (FA) and fiber length (FL) (Negwer et al., 2017a) have been established. The feasibility of these parameters in visualization of the most crucial language pathways (Sollmann et al., 2016), and their superiority to anatomical fiber tracking (Raffa et al., 2016; Negwer et al., 2017b) have been proven. Moreover, output maps have demonstrated their usefulness in the neurosurgical workflow (Sollmann et al., 2017a, 2018a): information through nTMS-based DTI-fiber tracking led to fewer deficits at discharge of neurosurgical patients operated on for resection of brain tumors, compared to a control group without nTMS-based DTI-fiber tracking (Raffa et al., 2017). Additionally, output maps have been found to increase the confidence of the neurosurgeon during the surgical procedure and enlarge the extent of safe resections (Sollmann et al., 2018a). Furthermore, it has been reported that this fiber tracking technique has the potential to capture the change in pre- to post-operative fiber count in relation to the drop in language scores (Ille et al., 2018; Negwer et al., 2018). In a case report, the method was proven to be feasible and useful, with high overlap in comparison with the gold standard for allocation of function through intraoperative mapping with Direct Electrical Stimulation (DES) (Sollmann et al., 2015a).

The influence of certain parameters on mapping results, such as choice of language error types induced by nTMS to use as ROIs, have been investigated (Sollmann et al., 2018b). Nevertheless, the impact of different language tasks when acquiring nTMS data seeds remains unexplored. Currently, the use of Object Naming to trigger noun retrieval is common for nTMS language mapping and subsequent tracking (Sollmann et al., 2015a, 2016, 2018a,b; Raffa et al., 2016, 2018; Negwer et al., 2017a, 2018; Ille et al., 2018). However, this contrasts with a more varied approach to testing using several tasks intra-operatively (Bello et al., 2006, 2007; Rofes and Miceli, 2014; De Witte et al., 2015a,b; Rofes et al., 2017). The question arises, therefore, if better visualization of fiber tracts could be achieved through implementation of a second picture naming task under nTMS-based DTI-fiber tracking, namely using Action Naming to trigger verb retrieval. This question becomes even more compelling, when the function of the different tracts to be visualized is taken into consideration. Although not yet perfectly delineated, the white matter pathways are thought to be differentially involved in cognitive processes that can consequently be interrogated to varying extent depending on the task (Rofes and Miceli, 2014; De Witte et al., 2015b). While Object and Action Naming are both believed to tap into visual picture recognition, lexico-semantic processing, phonological retrieval, and articulation planning and execution (Chang et al., 2015), it has been argued that Action Naming requires more cognitive resources to implement these processes (Bastiaanse and Van Zonneveld, 2004; Bastiaanse et al., 2016). Especially when used with a carrier phrase, Action Naming triggers embedding of a verb into a sentence, and, thus, demands more resources at grammatical, conceptual, and lexico-semantic stages (Rofes and Miceli, 2014; Ohlerth et al., 2020). In combination with the assumption of an, at least partially, segregated neural network subserving retrieval of verbs and nouns (Vigliocco et al., 2011; Crepaldi et al., 2013), it is reasonable to assume that seeds for tractography based on language mapping using Action Naming are different from those derived from mapping using Object Naming, particularly when considering the dual stream system of language processing in the white matter of the brain.

Classically, a ventral stream consisting of at least the Inferior Fronto-Occipital Fascicle (IFOF), the Uncinate Fascicle (UF), and potentially the Inferior Longitudinal Fascicle (ILF) is thought to support semantic processing, whereas a dorsal stream, consisting of the Arcuate Fascicle (AF) and the Superior Longitudinal Fascicle (SLF), is considered to implement phonological processing and decoding (Hickok and Poeppel, 2004, 2007; Ueno et al., 2011; Friederici, 2012, 2015; Hickok, 2012; Chang et al., 2015). However, more recent data from stimulation mapping using Object Naming under intraoperative DES and nTMS-based DTI-fiber tracking suggest that these function specifications are not always as clear-cut. Specifically, under DES, the IFOF has been shown to be involved in elementary semantic processing (Duffau et al., 2005; Leclercq et al., 2010; Maldonado et al., 2011a; Chang et al., 2015; Friederici, 2015), consistently leading to semantic paraphasias (Duffau et al., 2005; Bello et al., 2007, 2008, 2010; Leclercq et al., 2010; De Witt Hamer et al., 2011; Maldonado et al., 2011a), as well as showing a high correlation between nTMS seeding areas for semantic paraphasias and subsequent visualization of the IFOF (Raffa et al., 2016). The UF’s involvement in semantics is less apparent. While it was predominantly semantic paraphasias that appeared under stimulation of the UF with DES (Duffau et al., 2005, 2009; Bello et al., 2007, 2008), the role of the UF in syntactic processes such as grammar learning (Chang et al., 2015; Friederici, 2015) is still disputed (Chang et al., 2015; Akinina et al., 2019).

Though anatomically considered part of the ventral stream, the role of the ILF in language processing is not yet fully understood. In recent theories, it is considered that the ILF is an indirect ventral route that takes over, together with the UF, when the direct route of the IFOF is impaired (Mandonnet et al., 2007; Duffau et al., 2013, 2014). However, studies report that under DES, stimulation of the ILF results not only in anomias and semantic paraphasias, but also in phonological and articulatory errors (Bello et al., 2007; Maldonado et al., 2011a; Moritz-Gasser and Duffau, 2013; Moritz-Gasser et al., 2013) as well as a breakdown of spontaneous speech (Bello et al., 2007). Moreover, visualization of the ILF using nTMS-based DTI-fiber tracking has been achieved with the use of semantic paraphasias as ROIs (Raffa et al., 2016). Hence, while the ILF appears to be involved in phonological encoding, its role in building semantically (Moritz-Gasser et al., 2013), grammatically and phonologically appropriate units (Bello et al., 2007; Friederici, 2015) is still debated (Mandonnet et al., 2007; Chang et al., 2015; Sollmann et al., 2018b; Herbet et al., 2019).

In the dorsal stream, the AF is generally thought to support phonological processing (Duffau et al., 2002, 2014; Catani and Ffytche, 2005; Maldonado et al., 2011a; Moritz-Gasser and Duffau, 2013). While DES investigations overall confirm this by typically reporting phonological and phonemic errors (Duffau et al., 2002; Bello et al., 2007; Leclercq et al., 2010; Maldonado et al., 2011a; Chang et al., 2015), anomias have been reported, alongside visualizations of the AF through semantic paraphasias under nTMS seeding (Bello et al., 2006, 2007; Raffa et al., 2016; Sollmann et al., 2018b). Thus, while the evidence overwhelmingly points toward phonological processing, semantic and anomic errors reflect that the AF is also engaged in semantic processing. Moreover, its role in grammatical learning should not be neglected (Chang et al., 2015; Friederici, 2015; Ries et al., 2019).

As the second component of the dorsal stream, the SLF’s involvement is defined more clearly as serving phonological encoding and articulation (Makris et al., 2005; Bello et al., 2007; Moritz-Gasser and Duffau, 2013; Duffau et al., 2014). The SLF’s impairment under DES is largely correlated with dysarthric errors in articulation processes (Leclercq et al., 2010; Maldonado et al., 2011b) and phonemic paraphasias (Bello et al., 2008). Moreover, visualization of the tract through phonemic and articulatory errors under nTMS-based DTI-fiber tracking was reported (Sollmann et al., 2018b).

Finally, the Corticonuclear tract (CNT), which connects the motor cortex with the brain stem, has been visualized under nTMS-based DTI-fiber tracking (Negwer et al., 2017a; Sollmann et al., 2018b). It is believed to enable speech motor functions and, hence, to be a relevant component of speech production regardless of which task is used.

In sum, because Action Naming requires a greater grammatical, conceptual and lexico-semantic processing load, this may result in better visualization of the ventral stream tracts IFOF, UF, and ILF, as well as the dorsal stream AF. Object and Action naming are expected to result in a similar distribution for tracts subserving phonological and phonemic processes such as the SLF and the CNT for speech motor function.

To test these hypotheses, the current study aimed to evaluate nTMS-based DTI-fiber tracking results made on the basis of ROIs through Object Naming or Action Naming (in sentence context) in healthy volunteers. As a primary comparison, existence of the main tracts was measured under the two tasks to assess their efficiency to visualize the tracts within the dominant left hemisphere (LH). As an exploratory secondary comparison, contralateral involvement of tracts in the right hemisphere (RH) was analyzed. The debate on the exact involvement of the right hemisphere in hosting *language-involved areas* as opposed to *language-eloquent areas* in the LH is still ongoing (Sollmann et al., 2014; Vilasboas et al., 2017; Ohlerth et al., 2021). To shed light on this and its implications, the current study also includes tracking of the RH pathways as well as commissural tracts, supporting communication between the two hemispheres. The presence of an overall higher fraction of tracts in the LH, compared to the RH was hypothesized. Moreover, the RH is said to participate in more subtle integration processes of language as well as bilateral conceptual-semantic processing (Beeman et al., 2000; Marini et al., 2005; Chang et al., 2011; Vilasboas et al., 2017; Rolland et al., 2018; Sarubbo et al., 2020), possibly more crucial for Action Naming in sentence context. The expectation, therefore, was to find more commissural and overall, more RH involvement at the subcortical level for Action compared to Object Naming.

## Materials and Methods

### Ethics

The study complied with the ethical standards of the local review board (registration number: 202/18 S) as well as the Declaration of Helsinki and its amendments. Before testing commenced, written informed consent was collected from all volunteers partaking in this study.

### Participants

A cohort of 18 native speakers of German with a mean age of 24.94 years (± 7.153, range: 20–53 years) participated in the study. 13 volunteers were female, and the mean Edinburgh Handedness Inventory (EHI) score was 74.44 ± 18.50 (range: 45–95) (Oldfield, 1971). Exclusion criteria were left-handedness, background of any neurological or psychiatric diseases, and contraindication to MRI or nTMS data acquisition.

### Procedure

#### MRI

Prior to nTMS and tractography, individual MRIs of the participants were obtained on a 3T MRI scanner (Achieva dStream or Ingenia; Philips Healthcare, Best, The Netherlands) with a 32-channel head coil, entailing a three-dimensional (3D) T1-weighted turbo field echo sequence without contrast agent (TR/TE: 9/4 ms, 1 mm^3^, isovoxel covering the whole head) and a DTI sequence (TR/TE: 5,000/78 ms, voxel size of 2 × 2 × 2 mm^3^, one volume at b = 0 s/mm^2^, 32 volumes at b = 1,000 s/mm^2^).

#### Navigated Transcranial Magnetic Stimulation

**FIGURE 1:**
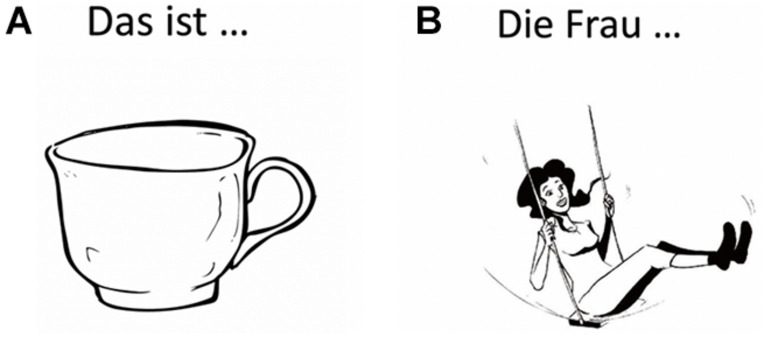
Example stimuli for **(A)** Object Naming (“Das ist eine… Tasse”: “This is a cup,” noun and article in German inflected for number, gender and case) and **(B)** Action Naming (“Die Frau… schaukelt”: “The woman swings,” verb in German inflected for person, number and tense). See (Ohlerth et al., 2020) for a detailed description of the task. (Artwork by Victor Xandri Antolin; University of Groningen).

Language mapping under nTMS has been described in detail previously (Krieg et al., 2017; Ohlerth et al., 2021). In brief, the T1-weighted turbo field echo sequence for each participant were transferred to the Nexstim eXimia NBS system (version 4.3; Nexstim Plc., Helsinki, Finland). The Verb And Noun test for Peri-OPerative testing (VAN-POP, Ohlerth et al., 2020) contains standardized tests for Object Naming and Action Naming in sentence context, triggering participants to produce short sentences with a target noun or target verb in its inflected form (see Figure 1). Two rounds of baseline naming were used per participant to allow elimination of stimuli that could not be named fluently and consistently by the participant.

Language mapping was conducted adopting the established protocol by Krieg et al. (2017), with the exception of a longer picture-presentation time of 1,000 ms. This choice was made as the picture stimuli in the VAN-POP are visually more complex than the default set of pictures provided by the NBS system. Following the Krieg et al. (2017) protocol entailed a 5 Hz/5 pulse stimulation of 10 rTMS trains per stimulation.

**FIGURE 2:**
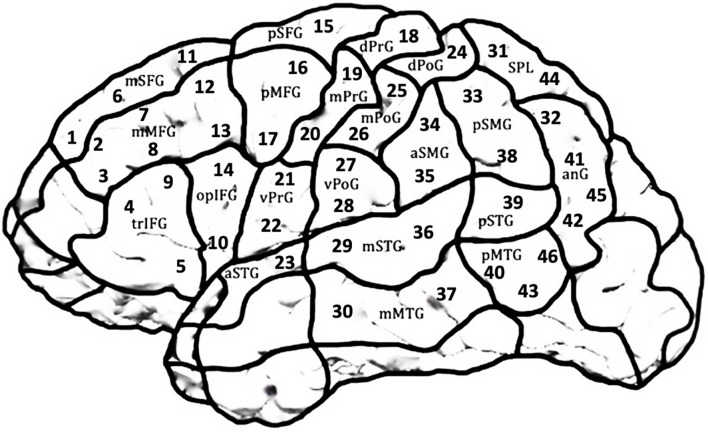
Scheme of the LH covering the 21 cortical parcellation system (CPS) regions consisting of 46 stimulation targets. The same regions were targeted in the RH in a mirror-inverted fashion. Due to high discomfort during stimulation, occipital areas, frontal and temporal poles and inferior temporal regions were excluded.

The two tasks were administered twice in blocks with randomized item order. A cortical area covered by 46 predefined stimulation points according to the Cortical Parcellation System (Corina et al., 2005; Figure 2) was tested in both hemispheres in a randomized order for both Object Naming and Action Naming. Each stimulation point was targeted 3 times over the 2 rounds for each task with a resting Motor Threshold (rMT) of 110% per participant, amounting to 6 stimulations per point and task.

#### Error Analysis

A *post hoc* video comparison of responses during the baseline testing against responses under stimulation was carried out. Responses during stimulation were classified as errors, if any of the following speech and language disruptions was observed: no response, hesitation on whole sentence, semantic paraphasia, anomia, hesitation on target, grammatical error, performance error, phonological error, and phonetic-articulatory error (Corina et al., 2010; Lioumis et al., 2012; Picht et al., 2013; Hernandez-Pavon et al., 2014; Krieg et al., 2017; Ohlerth et al., 2021). These error occurrences were considered to reveal language-positive cortical spots and their respective location was marked on the DICOM. Separate DICOM exports with error locations were computed per hemisphere and per task for Object and Action Naming.

#### Tractography Through Navigated Transcranial Magnetic Stimulation-Based Diffusion Tensor Imaging-Fiber Tracking

A deterministic tracking algorithm as implemented in the Brainlab iPlan Net Server (version 3.1.0.61; Brainlab AG, Munich, Germany) was applied to separately delineate subcortical tracts of the LH and RH and for Object and Action Naming. In this regard, both the DTI and T1-weighted sequences were planned with a field of view covering the whole head and co-registered to each other, thus allowing tractography for the whole brain. Then, nTMS-based tractography was carried out using solely the language-positive nTMS points of the LH and RH as derived from nTMS-based language mapping during the two tasks, respectively, without adding additional or manually drawn ROIs.

The language-positive stimulation points were co-registered to the MRI dataset of the respective volunteer, and eddy current correction was carried out. For task ROI creation, the language-positive nTMS spots, represented as three-dimensional objects in a column of three points at 0, 5, and 10 mm from the cortical surface, were exported from the NBS system and transferred to cubes: By adding 5 mm rims to each error spot, this resulted in cube-shaped ROIs with 3 × 3 × 3 voxels (Sollmann et al., 2016; Negwer et al., 2017a). If several errors occurred in one CPS region, only the first ROI within this region was kept and the remaining eliminated in order to not increase the size of the ROI per CPS region.

An individualized approach was used to identify the FA threshold (FAT) per participant: Starting with a FL of 110 mm, the FA was increased stepwise until no fibers were displayed. After decreasing the FA by 0.01, this value was then established as the individual FAT (Frey et al., 2012; Sollmann et al., 2016) and henceforth used. Per participant, the following adjustments were assessed for fiber visualization with a FL of 100 mm: FA = 0.1/0.15/50% of FAT/75% of FAT. Tracking adjustments leading to the highest fraction of overall tract visualization among all participants were established as 50% of the individual FAT and were used for further analysis (Sollmann et al., 2018b).

**FIGURE 3:**
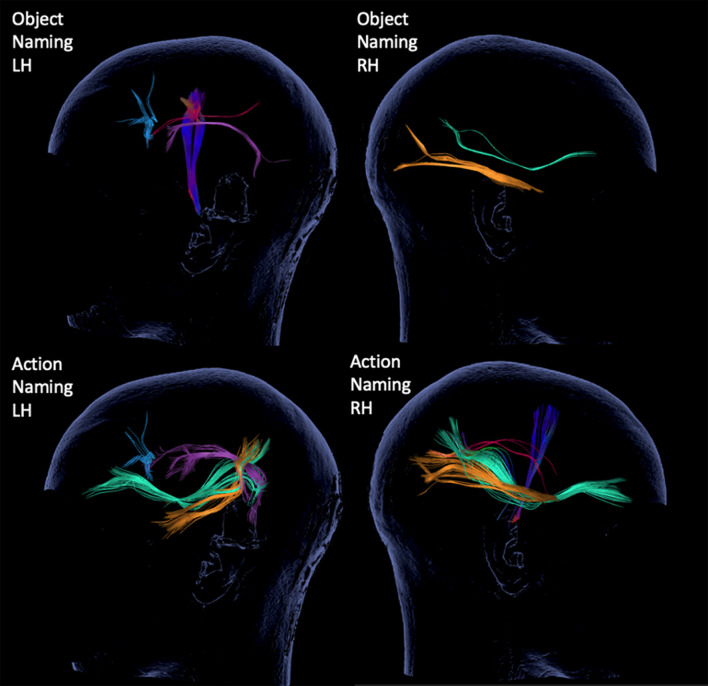
3D tractography results for each of the tests and hemispheres from representative case P7. The inspected tracts are color-coded as follows: AF in purple, SLF in red, ILF in yellow, IFOF in green, CF in blue and CNT with direction defined coloring, here in dark blue.

The resulting 3D displays of tractography were evaluated for existence of seven subcortical tracts (Catani and Thiebaut de Schotten, 2008; Kuhnt et al., 2012; Axer et al., 2013; Gierhan, 2013; Chang et al., 2015; Negwer et al., 2017a): AF, SLF, ILF, UF, IFOF, CNT, and inter-hemispheric commissural fibers (CF). The tracts were color-coded, as seen in the 3D tractography of representative case P7 (Figure 3).

### Statistical Analysis

R software (version 3.5.2; The R Foundation for Statistical Computing, Vienna, Austria) was used to generate descriptive statistics for age, handedness, rMT per hemisphere, baseline errors per task, and errors under nTMS for each task and hemisphere. Wilcoxon-Mann-Whitney tests were used to compare rMT in the LH vs. RH, and to compare baseline errors and errors under nTMS between Object and Action Naming per task and hemisphere.

For tractography results based on task ROIs of Object Naming or Action Naming per participant, the presence or absence of the seven tracts was noted, together with the respective tract volumes. To disentangle the language network from the speech-motor network, the CNT was analyzed separately and the remaining six tracts were analyzed individually as well as grouped together as “all language tracts.”

Proportions of participants showing tracts were compared: LH Object Naming task ROIs against Action Naming task ROIs; RH Object naming task ROIs against Action Naming task ROIs; all task ROIs in the RH against all task ROIs in the LH. In order to compare the frequency of successful visualization of tracts under the different task ROIs, Barnard’s tests were applied.

As an evaluation of the secondary factors *error rate* and *baseline errors* as predictors for existence of single tracts, logistic regression modeling was used with existence of tract as outcome variable and *task ROIs* (Object Naming vs. Action Naming), *baseline errors*, and *error rate* during nTMS as predictors.

## Results

All 18 participants tolerated nTMS mapping well and administration led to error elicitation in both tasks in all participants. There were no reports of fatigue or added difficulty by either of the tasks. No mapping block had to be excluded. The entire mapping procedure including registration to neuro-navigation, motor threshold hunting and mapping with both tasks took 2 h per participant. The rMT in the LH (35 ± 6.24, range: 25–50) and the RH (33.89 ± 6.42, range: 22–47) did not differ significantly (*p* = 0.431). Baseline errors during Action Naming (11.11 ± 4.47, range: 4–25) were significantly higher than baseline errors during Object Naming (3.44 ± 2.12, range: 1–8; *p* < 0.001).

Error rates during nTMS, FAT and used FA of 50% are given in Table 1 per task and hemisphere. In both hemispheres, significantly more errors were elicited during Action Naming than Object Naming LH: *p* = 0.024; RH: *p* = 0.021). FA values did not differ significantly (FAT: LH: *p* = 0.649; RH: *p* = 0.074; FA used: LH: *p* = 0.513; RH: *p* = 0.196). Between hemispheres, no comparison was significant for either error rate or any FA value (error rate ON: *p* = 0.361; AN: *p* = 0.206; FAT ON: *p* = 0.363; AN: *p* = 0.170; FA used ON: *p* = 0.361; AN: *p* = 0.406).

**TABLE 1:** Mapping and tracking parameters per task and hemisphere in mean ± standard deviation.

	**Error rate**	**FA threshold**	**FA used**
Object Naming in LH	11.78 ± 7.66	0.36 ± 0.06	0.18 ± 0.03
Object Naming in RH	12.66 ± 9.26	0.34 ± 0.08	0.17 ± 0.04
Action Naming in LH	16.05 ± 6.41	0.34 ± 0.06	0.17 ± 0.03
Action Naming in RH	17.33 ± 7.61	0.37 ± 0.07	0.19 ± 0.03

### Comparison of Visualized Tracts

All tracts could be visualized in at least some of the participants, apart from the UF which was not visualized in any participant. Thus, the UF was excluded from the next steps of the analysis. Table 2 shows the fraction of participants in whom the tracts could be visualized based on the task ROIs for Object Naming and Action Naming and their statistical comparison using Barnard’s tests.

**TABLE 2:** Percentages of participants with visualized tracts (with Barnard’s test comparison between task ROIs in the LH and RH).

**Tract**	**ROIs Object Naming in LH**	**ROIs Action Naming in LH**	**Comparison Barnard’s *p*-value**	**ROIs Object Naming in RH**	**ROIs Action Naming in RH**	**Comparison Barnard’s *p*-value**
CNT	66.7%	72.2%	0.827	83.3%	94.4%	0.351
AF	50%	55.6%	0.868	38.9%	38.9%	0.999
SLF	33.3%	38.8%	0.840	38.9%	44.4%	0.848
ILF	50%	83.3%	0.038*	55.6%	55.6%	0.999
IFOF	61.1%	100%	0.004*	61.1%	88.8%	0.066
CF	38.8%	38.8%	0.999	22.2%	33.3%	0.527
All language tracts	46.6%	63.3%	0.026*	43.3%	52.2%	0.255

*Use of bold font and an asterisk indicates statistical significance.*

For the comparison between hemispheres, Table 3 shows the fraction of participants in whom the tracts could be visualized based on the ROIs of the two tasks in the LH vs. the RH and their statistical comparison.

**TABLE 3:** Percentages of participants with visualized tracts (with Barnard’s test comparison between hemispheres for ROIs in the LH and RH).

**Tract**	**ROIs Object Naming in LH**	**ROIs Object Naming in RH**	**Comparison Barnard’s *p*-value**	**ROIs Action Naming in LH**	**ROIs Action Naming in RH**	**Comparison Barnard’s *p*-value**
CNT	66.6%	83.3%	0.283	72.2%	94.4%	0.105
AF	50%	38.9%	0.590	55.5%	38.9%	0.392
SLF	33.3%	38.9%	0.840	38.8%	44.4%	0.848
ILF	50%	55.5%	0.868	83.3%	55.6%	0.141
IFOF	61.1%	61.1%	0.999	100%	88.8%	0.202
CF	38.8%	22.2%	0.316	38.8%	33.3%	0.840
All language tracts	46.6%	43.3%	0.752	63.3%	52.2%	0.141

### Logistic Regression

Logistic regression modeling (see Table 4) for the LH data showed that *task ROI* was a significant predictor for visualization of tracts (Table 4, *z* = -2.326, *p* = 0.020), but neither *error rate* nor *baseline error rate* reached significance. For the RH data, *error rate* but not *task ROI* was a significant predictor for visualization of tracts (Table 4, *z* = 2.117, *p* = 0.034).

**TABLE 4:** Logistic regression model output for the LH and RH.

	**LH**				**RH**			
		
	**Estimate**	**SE**	***z*-value**	***p*-value**	**Estimate**	**SE**	***z*-value**	***p*-value**
Intercept	1.04	0.57	1.83	0.068	0.51	0.56	0.91	0.362
Task ROI	**−1.00**	**0.43**	**−2.33**	0.020*	**−**0.72	0.43	**−**1.68	0.093
Baseline error rate	**−**0.06	0.04	**−**1.38	0.167	**−**0.07	0.04	**−**1.69	0.092
Error rate	0.01	0.02	0.66	0.514	**0.036**	**0.02**	**2.12**	0.034*
AIC	295.68				297.72			

*Use of bold font and an asterisk indicates statistical significance.*

## Discussion

The purpose of the current study was to determine the relative potential of Object and Action Naming tasks to visualize the known subcortical language network, using the relatively novel method of nTMS-based DTI-fiber tracking. This is the first study to explore the use of a task other than Object Naming for ROI seeding for nTMS-based DTI-fiber tracking. By using a standardized and linguistically delineated Action Naming task (Ohlerth et al., 2020), this study takes the first step toward shedding light on the potential contribution of distinct ROIs for DTI-fiber tracking, using different language tasks. Moreover, the data allow conclusions to be drawn regarding the subcortical organization of the language network in the non-dominant RH as well as exploring the presence of inter-hemispheric pathways.

In the analysis of the network of the dominant LH, overall a higher fraction of language tracts was visualized when employing ROIs from the verb task, Action Naming, compared to employing ROIs from the noun task, Object Naming. Conversely, the fraction of speech pathways visualized, as measured in the CNT, connecting speech motor cortices with the corticospinal tract to eventually evoke muscle movement, did not differ between Action Naming and Object Naming ROIs. This contrast is in accordance with our initial hypothesis: while the CNT is involved in *speech* motor processing regardless of the task, the choice of task has an effect on visualization of *language* processing tracts. That is, the errors resulting from the more complex Action Naming task when used as ROIs may lead to more robust network visualizations than the Object Naming ROIs. This finding was confirmed both by Barnard’s test and further by logistic regression modeling, where the model showed no significant effect of the *quantity* of errors as measured in the error rate, but underlining that the *type* of errors (Object Naming vs. Action Naming) predicted visualization of tracts. Whereas this may seem a trivial assumption, it underlines the crucial difference between Object Naming and Action Naming. Moreover, this finding falls in line with cortical mapping results both with nTMS and intraoperative DES, which point toward the benefit of including verb tasks in mapping (Corina et al., 2005; Lubrano et al., 2014; De Witte et al., 2015b; Havas et al., 2015; Rofes et al., 2017; Ohlerth et al., 2021). While revealing distinct language areas during cortical mapping, here we show that verb tasks also contribute differently to exposing language involvement at the subcortical level. As we hypothesized that this contribution may differ depending on the pathways in question, we performed separate analyses for the dorsal and ventral streams including their respective single tracts.

### Ventral Stream

The ventral stream is commonly characterized as supporting lexico-semantic processing by connecting visual input areas to both temporal and frontal areas that are thought to be engaged in lexical retrieval (Hickok and Poeppel, 2004, 2007; Ueno et al., 2011; Friederici, 2012, 2015). Therefore, we hypothesized that the ventral stream would be more frequently visualized by Action Naming ROIs. This was based on the assumption that verb production in sentence context requires a higher processing load during conceptual and lexico-semantic retrieval and grammatical processes than noun production (Bastiaanse and Van Zonneveld, 2004; Rofes and Miceli, 2014; Bastiaanse et al., 2016; Ohlerth et al., 2020). Errors resulting from disturbance of Action Naming with nTMS were, therefore, hypothesized to be more likely potent for visualizing the lexico-semantic stream. Our results support this hypothesis by showing that more visualizations of the ventral stream pathways were achieved based on Action Naming ROIs than on Object Naming ROIs for both the IFOF and the ILF.

The IFOF is the most clearly delineated language pathway to consistently lead to lexico-semantic errors under DES (Duffau et al., 2005; Bello et al., 2007, 2008, 2010; Leclercq et al., 2010; De Witt Hamer et al., 2011; Maldonado et al., 2011a), and has also been visualized based on semantic errors under nTMS (Raffa et al., 2016). It is considered the direct route of the ventral stream, being primarily involved in lexico-semantic processing (Duffau et al., 2013, 2014). Visualized in 100% of participants in the current study through Action Naming in the LH and only in 61.1% through Object Naming, it underlines the potential of the lexico-semantically demanding Action Naming task in the setup at hand.

The same hypothesis holds for the ILF, which in combination with the UF establishes the indirect ventral route and is argued to take over in case of a damaged IFOF (Duffau et al., 2013, 2014): The ILF was visualized in 83.3% of cases under Action Naming which was significantly more than under Object Naming (50%). Its functional role has not been fully specified, with previous studies reporting an array of error types associated with stimulation of the ILF. Some studies have even claimed that the ILF is not related to language processing at all (Bello et al., 2007; Mandonnet et al., 2007; Maldonado et al., 2011a; Duffau et al., 2013, 2014; Raffa et al., 2016). This claim is not supported by the data in our study, as the ILF was frequently visualized under ROIs following nTMS disruption with both tasks. Furthermore, the significant difference in favor of Action Naming suggests a strong role for the ILF in lexico-semantic processing.

The second component of the indirect ventral route, the UF, could not be visualized at all in this study, possibly due to its very anterior temporal and frontal endpoints that are hard to reach by nTMS mapping. Administration of nTMS to these regions leads to high discomfort through peripheral nerve and muscle stimulation in the vicinity and, therefore, these regions are often omitted in current mapping protocols, as they were in our study. Although some nTMS-based DTI-fiber tracking studies have previously reported visualization of the UF (Negwer et al., 2017a; Sollmann et al., 2018b), all employed a rather broad range of tracking parameters, most importantly low reaching fractional anisotropy values that are likely to overrepresent fibers (Negwer et al., 2017b). The current protocol refrained from using FA values of lower than 0.1. This discrepancy may account for the lack of findings for the UF in this study.

### Dorsal Stream

The dorsal stream is overall believed to subserve phonological processing (Ueno et al., 2011; Friederici, 2012, 2015; Hickok, 2012). Consequently, our predictions did not entail a systematic difference in visualization of the dorsal stream pathways between Object Naming and Action Naming with the exception postulated for the AF. As the AF has been described as a component of grammatical processing (Chang et al., 2015; Friederici, 2015; Ries et al., 2019), we therefore hypothesized that Action Naming ROIs may lead to higher visualization of the AF due to the grammatical processing load required when inserting an inflected verb in a sentence context. This operation is considered more complex compared to that of the noun task, and, hence, was hypothesized to potentially induce a more pronounced visualization of the AF under Action Naming compared to Object Naming. However, contrary to this specific prediction for the AF, but confirming the overall hypothesis related to the dorsal stream, no difference in visualization was found between the two tasks for the AF and SLF. This comparable distribution lends support to the phonological involvement of the dorsal stream, which could be seen regardless of the specific task used.

### Right Hemisphere

Mapping language in the RH has long played a subordinate role. While on the one hand, partial involvement of the RH regions during language tasks is suggested by several imaging studies (for a review, see Crepaldi et al., 2011), on the other, minor to no language impairments have been reported after RH lesions or their resection, leading to the initial assumption that the RH merely plays a secondary, non-essential role in language (Duffau et al., 2003). The RH may only be crucial in left-handers with a less predictable lateralization or as a compensator after LH damage (Duffau et al., 2008). This view of a strong left lateralization has been challenged following more in-depth language assessment after stroke, lesion mapping, and resection in the RH, indicating the presence of language deficits (Boller, 1968; Bryan, 1988; Joanette et al., 1988; Thompson et al., 2016; Antonsson et al., 2018; Gajardo-Vidal et al., 2018). Mapping results with nTMS underline this reevaluation, as nTMS evoked a similar amount of language errors in the RH and the LH in healthy volunteers and was able to capture the shift of language to the RH in tumor patients with a left-hemispheric lesions (Krieg et al., 2013; Rösler et al., 2014; Sollmann et al., 2014, 2015b; Ille et al., 2016). Moreover, the latest addition of opposing data to overcome the view of a linguistically irrelevant RH stems from intraoperative DES of the RH itself. Positive language sites have in recent years been reported not only in left-handers, but also in ambidextrous and right-handed individuals, leading to the proposal of an entire connectome of language organization in the RH with close to all subfunctions of language in a homotopic organization to that of the LH (Vassal et al., 2010; Chang et al., 2011; Tate et al., 2014; Vilasboas et al., 2017). Importantly, this language connectome is based exclusively on verbal and non-verbal tasks for object semantics, but nonetheless resembles the known LH counterparts: a RH ventral stream is assumed that mirrors the LH concept, both in structural connectivity (De Benedictis et al., 2014; Ruschel et al., 2014) and in function of conceptual, non-verbal semantic processing (Duffau et al., 2008; Herbet et al., 2017; Vilasboas et al., 2017; Rolland et al., 2018; Sarubbo et al., 2020). Taken together with data from the LH, a bilateral multi-modal semantic network with a LH hemisphere verbal and RH non-verbal lateralization has been proposed, after reporting errors during verbal Object Naming and non-verbal noun tasks of stimulation of the IFOF in the LH and RH, respectively (Vilasboas et al., 2017; Rolland et al., 2018; Sarubbo et al., 2020).

The dorsal stream’s main function in phonological processing, typically seen in induced phonological errors through stimulation of the AF, is mostly found in the LH (Silva and Citterio, 2017; Sarubbo et al., 2020). Recently, phonemic errors induced by stimulation of the right SLF led authors to hypothesize a parallel partial dorsal stream for phonological to articulatory computing in the RH (Vilasboas et al., 2017), and not only in left-handers (Duffau et al., 2008). The most commonly observed assumption is that of a bihemispheric articulatory-motor loop: articulatory disturbances related to the right SLF and CNT are in line with the speech motor system controlled by a bilateral circuit (Vilasboas et al., 2017; Rolland et al., 2018; Sarubbo et al., 2020). Consequently, more systematic mapping of the RH’s speech and language areas is recommended and establishes the need for non-invasive approaches like nTMS language mapping as well. In this context, it is important to keep in mind that, while being the reference standard for functional brain mapping, DES does not allow for bilateral mapping within the same individual because it is spatially restricted to the area of exposed cortex and subcortical white matter as determined by the borders of the craniotomy. Hence, it cannot inform the debate on dominance by contributing data on bihemispheric language representations on single-subject level. However, nTMS can be applied to both hemispheres in the same individual leading to bihemispheric virtual lesion mapping and, thus, offers a unique way to interrogate the brain supplementing DES.

In the view of previous data from DES, fMRI, and behavioral studies, we expected to be able to visualize a less pronounced network in the RH compared to the LH under nTMS language mapping. However, the inter-hemispheric comparison yielded no significant difference. The overall fraction of white matter pathways that could be visualized in the RH did not differ from the left hemisphere. Our data, hence, do not support classical LH language dominance, but rather fall in line with the DES data from righthanders (Vilasboas et al., 2017). Our findings strengthen the overall claim from the intraoperative data and through non-invasive data from healthy individuals, suggesting a systematic presentation of language in the RH similar to the LH.

Regarding the linguistic difference between the tasks and the abovementioned function allocations of the RH pathways, one may have expected an advantage from the grammatically, conceptually and lexico-semantically more demanding task of Action Naming in the RH, especially in the ventral stream. This was not the case. The previously claimed non-verbal semantic right lateralization was based on an object semantic task (non-verbal Pyramid and Palm Trees Test; Howard and Patterson, 1992) under DES (Rolland et al., 2018; Sarubbo et al., 2020). In this task, the participant is asked to choose the picture candidate that is associated to the target, without requiring lexical retrieval of the word form and its articulation. It remains unknown whether the conceptual and semantic requirements of Action Naming would have benefitted the visualization of the RH IFOF, had we administered two non-verbal tasks.

Overall, our results suggest that the RH ventral stream is as much involved in both naming tasks as its LH counterpart. Predictions differed for the dorsal stream: with a mostly leftward lateralization for phonological processing, a less visualized RH fraction would be expected, however, with no significant difference between tasks. Regardless of the noun or verb semantics, the two naming tasks do not require different processing loads for phonological to phonetic conversion. Hence, one would expect no inter-task difference, but only an inter-hemispheric difference. The first prediction held true with no significant differences between tasks. However, the visualization of the dorsal stream was just as successfully achieved in the RH as in the LH. This provides support to an entirely bilateral network for phonological-articulatory processes, as recently suggested (Vilasboas et al., 2017).

### Inter-Hemispheric Connections Through the Corpus Callosum

The corpus callosum as the largest white matter bundle of the human brain is known to enable communication between the two hemispheres. In the lesioned brain, these commissural fiber connections become particularly important as a mediator for reorganization: plasticity effects of the contralateral hemisphere may take over function of the ipsilateral hemisphere, and this shift is aided by increased corpus callosum involvement (Pravatà et al., 2014; Liu et al., 2015; Sollmann et al., 2017b). Little is known about the individual involvement of these tracts during language processing of specific tasks. A handful of studies report a correlation between poor performance on verb tasks and corpus callosum integrity (Riccardi et al., 2019), with no such relation to performance during Object Naming (Tomasino et al., 2019; Dresang et al., 2020).

Once more, the overall higher grammatical, conceptual, and lexico-semantic efforts of Action Naming in combination with previous findings lead to the hypothesis of more inter-hemispheric tracts visualized under nTMS-based DTI-fiber tracking for Action Naming in the present data. This prediction was not confirmed. Indeed, commissural fibers were shown in only 38% of cases, with no difference between tasks. The proposed psycholinguistic dissimilarities are, hence, not evident in the distribution of inter-hemispheric connections via the corpus callosum. Accordingly, this implies that based on nTMS disturbance and subsequent tracking of the subcortical language network, both tasks rely equally on these connections to support bilateral involvement, and both in a limited matter compared to intra-hemispheric pathways.

### Implications for Fiber Tracking in the Clinical Population

The method of nTMS-based DTI-fiber tracking has been developed specifically for individual fiber tracking in neurosurgical patients about to undergo resection within or close to potentially functional eloquent areas. Recently, our group has described their experience of applying a dual-task protocol, as used in the current study, in seven neurosurgical cases and has reported high applicability in the clinical workflow (Ohlerth et al., subm.). Adding one round of Action Naming in one hemisphere required a mere 15 min more compared to the standard mapping procedure of roughly 50 min, and can be considered a feasible approach from a practical point of view. Regarding the added value of the second task, the findings of the current study are, hence, specifically useful for this population, and have the following implications.

Firstly, it was shown that using the task Action Naming under nTMS with subsequent tracking leads to an overall more comprehensive network visualization of the known language tracts. Hence, preoperative mapping in patients may profit from using this task as well. Individuals with lesions invading the subcortical areas of the IFOF and ILF may benefit from Action Naming mapping followed by tractography using the obtained data for ROI seeding in particular. Successful visualization of these ventral stream tracts in relation to the tumor may help prevent lexico-semantic processing deficits after resection. Secondly, as also proposed by Vilasboas et al. (2017), preoperative nTMS RH mapping should be considered for patients suffering from a RH tumor, especially when minor impairments of language are already present and point toward a rightward lateralization in the individual, and when a high level of cognition is to be preserved. Thirdly, mapping and tracking with both tasks may become particularly crucial in cases of pronounced cortical and subcortical reorganization induced by tumor growth. The protocol at hand would allow detection of shifted functional loci not only for Object Naming, but also for the seemingly partly segregated Action Naming skill. Visualization of both skills, therefore, may benefit presurgical planning and risk stratification.

### Limitations

Inevitably, this study has limitations. DTI investigations, as delineated in our study, come with known disadvantages such as their incapability to address intra-voxel calculations, including crossing, kissing, branching and fanning fibers (Wiegell et al., 2000; O’Donnell and Westin, 2011). Moreover, with several available methods how to approach fiber tracking, the current setup was based on the clinically most common and useful protocol employing language-positive points derived from nTMS language mapping. This entails the careful administration of various fiber tracking parameters such as choice of angulation, FL, FAT and choice of computing algorithm. This means that the current findings may not be generalizable, if these parameters differ. Nor should the parameters be considered the only possible way to conduct fiber tracking. Nevertheless, the results of the method described in this study, as it is currently used in clinical practice, can benefit from the task choice presented here.

Furthermore, no error type analysis was carried out for relating specific tracts to specific functions. This was beyond the aim of the current study, which set out to compare the overall task of Action Naming against the commonly used Object Naming task. Despite its exploratory nature, the findings allow insight into the assumed overall functions of the tracts and streams. Furthermore, this approach remains closest to the clinical setting, which often precludes extensive error type analysis before tracking due to time-limiting factors.

Lastly, apart from one single case report, nTMS-based DTI-fiber tracking for language-involved tracts is still lacking ultimate validation by comparing its findings to subcortical language mapping through intraoperative DES in a group study (Sollmann et al., 2015a). Investigating a healthy cohort not undergoing surgery in the current study did not allow us to draw this comparison. Future research is needed to substantiate the usefulness and reliability of this method in a group of clinical cases.

## Conclusion

Administering an Action Naming task for nTMS-based DTI-fiber tracking not only enriches the depth of language testing, but improves the visualization of the language network in the LH, particularly for the left IFOF and ILF. This promising result urges the inclusion of this task in prospective presurgical mapping. Moreover, a closely mirrored network for language was detected in the RH, and calls for rethinking of its underrepresented role in language.

## Data Availability Statement

The raw data supporting the conclusions of this article will be made available by the authors, without undue reservation.

## Ethics Statement

The studies involving human participants were reviewed and approved by the Institutional Review Board of the Technical University of Munich. The patients/participants provided their written informed consent to participate in this study.

## Author Contributions

A-KO, RB, NS, and SMK contributed to the conception and design of the study. A-KO, CN, NS, SS, and AS performed the data collection and analysis. A-KO wrote the manuscript, with contributions and editing by all authors.

## Conflict of Interest

The authors declare that the research was conducted in the absence of any commercial or financial relationships that could be construed as a potential conflict of interest.

## Publisher’s Note

All claims expressed in this article are solely those of the authors and do not necessarily represent those of their affiliated organizations, or those of the publisher, the editors and the reviewers. Any product that may be evaluated in this article, or claim that may be made by its manufacturer, is not guaranteed or endorsed by the publisher.
